# Impact of obesity on cervical ossification of the posterior longitudinal ligament: a nationwide prospective study

**DOI:** 10.1038/s41598-022-12625-3

**Published:** 2022-05-25

**Authors:** Kanji Mori, Toshitaka Yoshii, Satoru Egawa, Kenichiro Sakai, Kazuo Kusano, Shunji Tsutsui, Takashi Hirai, Yu Matsukura, Kanichiro Wada, Keiichi Katsumi, Masao Koda, Atsushi Kimura, Takeo Furuya, Satoshi Maki, Narihito Nagoshi, Norihiro Nishida, Yukitaka Nagamoto, Yasushi Oshima, Kei Ando, Hiroaki Nakashima, Masahiko Takahata, Hideaki Nakajima, Kazuma Murata, Masayuki Miyagi, Takashi Kaito, Kei Yamada, Tomohiro Banno, Satoshi Kato, Tetsuro Ohba, Satoshi Inami, Shunsuke Fujibayashi, Hiroyuki Katoh, Haruo Kanno, Hiroshi Taneichi, Shiro Imagama, Yoshiharu Kawaguchi, Katsushi Takeshita, Morio Matsumoto, Masashi Yamazaki, Atsushi Okawa

**Affiliations:** 1 Department of Orthopaedic Surgery, Shiga University of Medical Science, Seta Tsukinowa-Cho, Otsu, Shiga 520-2192 Japan; 2 Department of Orthopedic Surgery, Tokyo Medical and Dental University, 1-5-45 Yushima, Bunkyo Ward, Tokyo, 113-8519 Japan; 3 Department of Orthopedic Surgery, Saiseikai Kawaguchi General Hospital, 5-11-5 Nishikawaguchi, Kawaguchishi, Saitama 332-8558 Japan; 4 Department of Orthopedic Surgery, Kudanzaka Hospital, 1-6-12 Kudanminami, Chiyodaku, 102-0074 Japan; 5 Department of Orthopaedic Surgery, Wakayama Medical University, 811-1 Kimiidera, Wakayama, 641-8510 Japan; 6 Department of Orthopedic Surgery, Hirosaki University Graduate School of Medicine, 5 Zaifu-cho, Hirosaki, Aomori 036-8562 Japan; 7 Department of Orthopedic Surgery, Niigata University Medical and Dental General Hospital, 1-754 Asahimachidori, Chuo Ward, Niigata, Niigata 951-8520 Japan; 8 Department of Orthopedic Surgery, Faculty of Medicine, University of Tsukuba, 1-1-1 Tennodai, Tsukuba, Ibaraki 305-8575 Japan; 9 Department of Orthopedics, Jichi Medical University, 3311-1 Yakushiji, Shimotsuke, Tochigi 329-0498 Japan; 10 Department of Orthopedic Surgery, Chiba University Graduate School of Medicine, 1-8-1 Inohana, Chuo Ward, Chiba, Chiba 260-0856 Japan; 11 Department of Orthopaedic Surgery, Keio University School of Medicine, 35 Shinanomachi, Shinjuku Ward, Tokyo, 160-8582 Japan; 12 Department of Orthopedic Surgery, Yamaguchi University Graduate School, of Medicine, 1-1-1 Minami-Kogushi, Ube, Yamaguchi 755-8505 Japan; 13 Department of Orthopedic Surgery, Osaka Rosai Hospital, 1179-3 Nagasonecho, Sakaishi, Osaka 591-8025 Japan; 14 Department of Orthopaedic Surgery, Faculty of Medicine, The University of Tokyo, 7-3-1 Hongo, Bunkyo-ku, Tokyo, 113-0033 Japan; 15 Department of Orthopedic Surgery, Nagoya University Graduate School of Medicine, 65 Tsurumaicho, Showa Ward, Nagoya, Aichi 466-8550 Japan; 16 Department of Orthopaedic Surgery, Faculty of Medicine and Graduate School of Medicine, Hokkaido University, Kita 15, Nishi 7, Sapporo, 060-8638 Japan; 17 Department of Orthopaedics and Rehabilitation Medicine, Faculty of Medical Sciences, University of Fukui, 23-3 Matsuoka Shimoaizuki, Eiheiji-cho, Yoshida-gun, Fukui 910-1193 Japan; 18 Department of Orthopedic Surgery, Tokyo Medical University, 6-7-1 Nishishinjuku, Shinjuku-ku, Tokyo, 160-0023 Japan; 19 Department of Orthopedic Surgery, Kitasato University, School of Medicine, 1-15-1 Kitazato, Minami-ku, Sagamiharashi, Kanagawa 252-0375 Japan; 20 Department of Orthopedic Surgery, Graduate School of Medicine, Osaka University, 2-2 Yamadaoka, Suita-shi, Osaka 565-0871 Japan; 21 Department of Orthopaedic Surgery, Kurume University School of Medicine, 67 Asahi-machi, Kurume-shi, Fukuoka 830-0011 Japan; 22 Department of Orthopedic Surgery, Hamamatsu University School of Medicine, 1-20-1 Handayama, Hamamatsu, Shizuoka 431-3125 Japan; 23 Department of Orthopaedic Surgery, Graduate School of Medical Sciences, Kanazawa University, 13-1 Takara-machi, Kanazawa, 920-8641 Japan; 24 Department of Orthopedic Surgery, University of Yamanashi, 1110 Shimokato, Chuo Ward, Yamanashi, 409-3898 Japan; 25 Department of Orthopaedic Surgery, Dokkyo Medical University School of Medicine, 880 Kitakobayashi, Mibu-machi, Shimotsuga-gun, Tochigi 321-0293 Japan; 26 Department of Orthopaedic Surgery, Graduate School of Medicine, Kyoto University, 54 Kawahara-cho, Shogoin, Sakyo-ku, Kyoto, 606-8507 Japan; 27 Department of Orthopedic Surgery, Surgical Science, Tokai University School of Medicine, 143 Shimokasuya, Isehara, Kanagawa 259-1193 Japan; 28 Department of Orthopaedic Surgery, Tohoku University School of Medicine, 1-1 Seiryomachi, Aoba Ward, Sendai, Miyagi 980-8574 Japan; 29 Department of Orthopedic Surgery, Faculty of Medicine, University of Toyama, 2630 Sugitani, Toyama, Toyama 930-0194 Japan; 30 Japanese Multicenter Research Organization for Ossification of the Spinal Ligament, Tokyo, Japan

**Keywords:** Outcomes research, Musculoskeletal system

## Abstract

Positive association between ossification of the posterior longitudinal ligament of the spine (OPLL) and obesity is widely recognized; however, few studies focused on the effects of obesity on treatment of cervical OPLL. The effects of obesity on surgical treatment of cervical OPLL were investigated by a Japanese nationwide, prospective study. Overall, 478 patients with cervical myelopathy due to OPLL were prospectively enrolled. To clarify the effects of obesity on the surgical treatment for cervical OPLL, patients were stratified into two groups, non-obese (< BMI 30.0 kg/m^2^) and obese (≥ BMI 30.0 kg/m^2^) groups. The mean age of the obese group was significantly younger than that of non-obese group. There were no significant differences between the two groups in other demographic information, medical history, and clinical and radiographical findings. Alternatively, the obese group had a significantly higher rate of surgical site infection (SSI) than that of non-obese group. Approach-specific analyses revealed that the SSI was significantly higher in the obese group than in the non-obese group. A logistic regression analysis revealed that age, BMI, and duration of symptoms were significant factors affecting the postoperative minimum clinically important difference success. The result of this study provides useful information for future cervical OPLL treatment.

## Introduction

Ossification of the posterior longitudinal ligament of the spine (OPLL), first described by Key in 1838^1^, is characterized by the heterotopic bone formation in the posterior longitudinal ligament of the spine. Cervical OPLL is more frequently confirmed in middle-aged and elderly patients and more common in men^2^. The etiology of OPLL is known to multifactorial with a genetic factor in addition to degenerative factors, such as mechanical stress and aging; however, it still remains elucidated^2^. Currently, OPLL is found not only in East Asia but also in other countries^3,4^. The prevalence of OPLL ranging from 0.1 to 2.5% in the United States and 1.9 to 4.3% in Japan^2,5^. OPLL can result in neurological compromises through compression of spinal cord and nerve roots^2^. Surgical decompression is recommended in cases characterized by progressive and/or severe myelopathy^6^. Surgical approaches include anterior, posterior, and anterior–posterior, which has unique risk and benefit, and are selected according to the case^7^. However, the optimal surgical procedure for the treatment of cervical OPLL remains to be established^7,8^.

The World Health Organization (WHO) defines obesity based on the body mass index (BMI)^9^. Obesity, defined BMI ≥ 30.0 kg/m^2^ is a major public health problem, with an estimated mortality of 3.4 million and 4% of disability-adjusted life-years worldwide^10^. Obesity has been associated with significant comorbidities, including heart disease, hypertension, stroke, diabetes mellitus, and obstructive sleep apnea^9,11^. All these not only increase the medical costs^12^, but also have negative effects on spine surgery, such as higher rate of postoperative complications^13–16^. There is increasing literature assessing the effects of obesity on thoracic and lumbar surgery, however limited literature assessing the effects of obesity on cervical spine surgery^17^.

So far, surgical outcomes for cervical OPLL have been reported, but there are few reports on perioperative complications^3,18^. In addition, although positive association between OPLL and obesity is widely recognized^19,20^; to the best of our knowledge, few studies have a comprehensive discussion focusing on the effects of obesity on the treatment of cervical OPLL.

The purpose of this study is to investigate the effects of obesity on surgical treatment of cervical OPLL including perioperative complications by a Japanese nationwide, prospective study.

## Results

**Figure 1:**
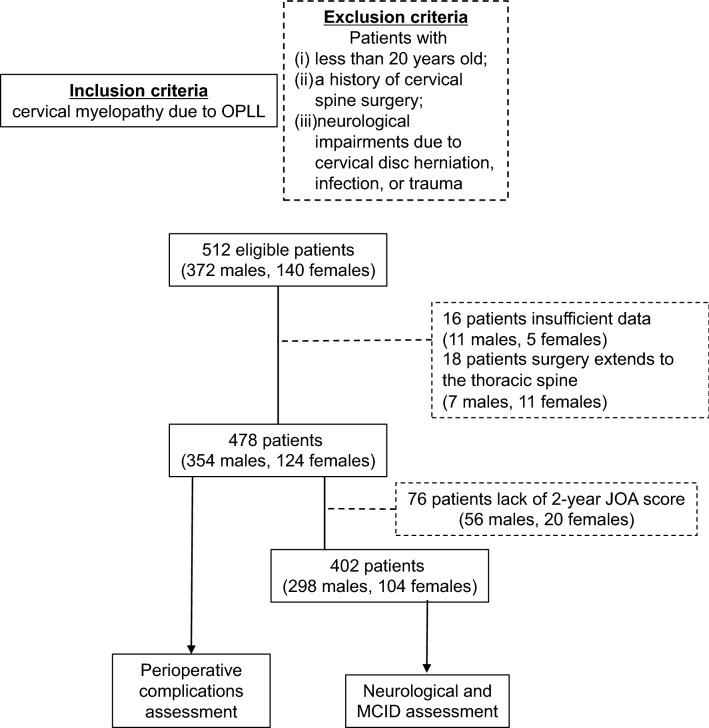
Flowchart of patients through the study. OPLL: ossification of the posterior longitudinal ligament; JOA score: Japanese Orthopedic Association score; MCID: minimum clinically important difference.

Flowchart of patients through the study is shown in Fig. 1. A total of 478 patients’ characteristics were summarized in Table 1. Seventy eight percent of all cases had at least one comorbidity and had a high frequency of hypertension (38.1%) and diabetes mellitus (30.8%). The non-obese group consisted of 413 patients and the obese group consisted of 65 patients. The mean ages of the non-obese and obese groups were 65.5 ± 11.0 years and 54.8 ± 11.1 years, respectively, and the obese group was significantly younger (p < 0.001). There were no significant differences between the two groups in comorbidities, smoking history, anticoagulant use, and duration of symptoms (Table 1). There was a significant difference in the surgical procedure selected between the non-obese and obese groups (p = 0.013). Laminoplasty was often selected in the non-obese group, on the other hand, anterior–posterior surgery was often selected in the obese group.

**Table 1:** Demographics of the participants.

	Non-obese (n = 413)	Obese (n = 65)	*p*-value
Gender (male) (%)	75.3	66.2	0.13
Age (yeas)	65.5 ± 11.0	54.8 ± 11.1	< 0.001
Comorbidity (%)	77.7	80.0	0.75
Diabetes mellitus (%)	29.5	38.5	0.15
Hypertension (%)	37.5	41.5	0.58
Malignancy (%)	5.3	0.0	0.57
Collagen disease (%)	1.0	0.0	1.00
Cerebrovascular event (%)	6.1	4.6	1.00
Myocardial infarction (%)	3.1	3.1	1.00
Smoking history (%)	35.1	47.7	0.072
Anticoagulant use (%)	16.0	15.4	1.00
Duration of symptoms (month)	43.2 ± 65.1	34.1 ± 36.9	0.10
**Surgical procedure**			0.013
ADF (%)	19.9	23.1	–
Anterior–posterior surgery (%)	1.9	6.2	–
Laminoplasty (%)	56.9	38.5	–
PDF (%)	21.3	32.3	–

Non-obese: BMI < 30.0 kg/m^2^; Obese: BMI ≥ 30.0 kg/m^2^; BMI: body mass index; ADF: anterior decompression and fusion; PDF: posterior decompression and fusion. Age and duration of symptoms were represented by mean ± standard deviation.

Radiographical assessments revealed that type of OPLL, C2-7 angle, C2-7 range of motion (ROM), K-line condition, and the presence of an intramedullary signal intensity change on T2-weighted magnetic resonance (MR) imaging were not significantly different between non-obese and obese groups (Table 2). The canal occupying ratio (COR) of the obese group (47.8 ± 17.8%) was high enough to be almost significantly different from that of the non-obese group (43.2 ± 15.4%) (p = 0.050).

**Table 2:** Radiographical features of non-obese and obese groups.

	Non-obese (n = 413)	Obese (n = 65)	*p*-value
**OPLL type**			0.33
Segmental (%)	37.8	30.8	–
Mixed (%)	42.4	50.8	–
Continuous (%)	12.6	15.4	–
Localized (%)	7.3	3.1	–
Thickness of ossification (mm)	5.50 ± 1.90	5.93 ± 2.02	0.098
COR (%)	43.2 ± 15.4	47.8 ± 17.8	0.050
C2-7 angle (degree)	10.3 ± 11.7	7.59 ± 11.1	0.085
C2-7 ROM (degree)	26.9 ± 14.0	24.9 ± 12.7	0.29
T2 high (%)	85.5	86.2	1.00
K-line (–) (%)	32.4	30.8	0.90

Non-obese: BMI < 30.0 kg/m^2^; Obese: BMI ≥ 30.0 kg/m^2^; BMI: body mass index; OPLL: ossification of posterior longitudinal ligament; COR: spinal canal occupying ratio; ROM: range of motion; T2 high: intramedullary high intensity area on T2-weighted images. Thickness of ossification, occupying ratio, C2-7 angle, and C2-7 ROM were represented by mean ± standard deviation.

Then, the perioperative complications of the non-obese and obese groups were investigated. There were no significant differences between the two groups in terms of neurological and general complications (Table 3). However, regarding local complications, the obese group had a significantly higher rate of surgical site infection (SSI) than the non-obese group. Subsequently, the effects of obesity on approach-specific perioperative complications were investigated. Patients’ demographic and radiographical data stratified by approach are shown in Tables 4 and 5, respectively. In anterior surgery, there were no significant differences between the non-obese and obese groups in all perioperative complications (Table 6). On the other hand, in posterior surgery, the frequency of SSI was significantly higher in the obese group (8.7%) than in the non-obese group (1.2%) (p = 0.010). No significant difference was found between the two groups for other perioperative complications (Table 6). By the way, there were 12 cases of anterior–posterior surgery, and no SSI was found in all cases. In addition, we also investigated the incidence of SSI in non-obese and obese groups with laminoplasty group alone and confirmed that obese group had a significantly higher incidence of SSI (1.5% vs. 9.1%, p = 0.023).

**Table 3:** Perioperative complications in non-obese and obese groups.

	Non-obese (n = 413)	Obese (n = 65)	*p*-value
Complications (%)	25.2	35.4	0.096
**Neurological**			
Quadriparesis (%)	1.69	1.50	1.00
Hemiparesis (%)	0.73	0.0	1.00
Paraparesis (%)	0.24	0.0	1.00
Upper extremity bil. (%)	0.24	1.5	0.25
Upper extremity uni. (%)	6.78	7.7	0.79
C5 palsy (%)	7.0	7.7	0.80
**Local**			
Dural tear (%)	4.12	9.23	0.11
CSF leakage (%)	1.94	1.50	1.00
Epidural hematoma (%)	0.48	0.0	1.00
Wound dehiscence (%)	0.73	1.50	0.45
Surgical site infection (%)	1.21	6.15	0.024
Graft complications (%)	1.94	0.0	0.61
**General**			
Dysphagia (%)	2.42	0.0	0.37
Upper airway obstruction (%)	0.48	1.50	0.36
PE/DVT (%)	0.48	1.50	0.36
Delirium (%)	1.69	1.50	1.00
UTI (%)	1.69	4.62	0.14

Non-obese: BMI < 30.0 kg/m^2^; Obese: BMI ≥ 30.0 kg/m^2^; BMI: body mass index; bil: bilateral; uni: unilateral; CSF: cerebrospinal fluid; PE: pulmonary embolism; DVT: deep venous thrombosis; UTI: urinary tract infection.

**Table 4:** Patients’ demographic data stratified by approach.

	Anterior (n = 97)			Posterior (n = 369)		
	Non-obese (n = 82)	Obese (n = 15)	*p*-value	Non-obese (n = 323)	Obese (n = 46)	*p*-value
Sex (male) (%)	68.3	66.7	1.00	77.1	67.4	0.20
Age (years)	61.8 ± 10.9	49.4 ± 9.94	< 0.001	66.7 ± 10.7	56.0 ± 11.1	< 0.001
Comorbidity (%)	67.1	66.7	1.00	80.5	82.6	0.84
Diabetes mellitus (%)	20.7	33.7	0.32	31.6	41.3	0.24
Hypertension (%)	30.5	40.0	0.55	39.9	41.3	0.87
Malignancy (%)	4.9	0.0	1.00	5.6	0.0	0.15
Collagen disease (%)	1.2	0.0	1.00	0.9	0.0	1.00
Cerebrovascular event (%)	2.4	0.0	1.00	7.1	6.5	1.00
Myocardial infarction (%)	4.9	0.0	1.00	2.8	4.3	0.63
Smoking history (%)	35.4	66.7	0.42	35.0	41.3	0.55
Anticoagulant use (%)	12.2	6.7	1.00	17.0	17.4	1.00
Duration of symptoms (m.)	50.1 ± 59.3	31.0 ± 36.1	0.23	41.2 ± 66.7	35.9 ± 38.6	0.60

Age and duration of symptoms were represented by mean ± standard deviation.

Non-obese: BMI < 30.0 kg/m^2^; Obese: BMI ≥ 30.0 kg/m^2^; BMI: body mass index.

**Table 5:** Patients’ radiographical data stratified by approach.

	Anterior (n = 97)			Posterior (n = 369)		
	Non-obese (n = 82)	Obese (n = 15)	*p*-value	Non-obese (n = 323)	Obese (n = 46)	*p*-value
**OPLL type**			0.16			0.66
Segmental (%)	41.5	13.3	–	37.5	37.0	–
Mixed (%)	37.8	60.0	–	42.7	45.7	–
Continuous (%)	11.0	20.0	–	13.0	15.2	–
Localized (%)	9.8	6.7	–	6.8	2.2	–
Thickness of ossification (mm)	6.05 ± 2.00	5.99 ± 1.96	0.90	5.33 ± 1.84	5.88 ± 2.14	0.061
COR (%)	49.6 ± 14.3	51.8 ± 18.8	0.61	41.3 ± 15.1	46.1 ± 17.7	0.053
C2-7 angle (deg.)	6.01 ± 11.0	5.67 ± 6.85	0.88	11.5 ± 11.7	8.62 ± 12.2	0.13
C2-7 ROM (deg.)	31.2 ± 14.9	30.0 ± 13.5	0.78	25.8 ± 13.6	22.7 ± 12.2	0.17
T2 high (%)	85.4	93.3	0.69	85.1	82.6	0.70
K-line (–) (%)	51.2	20.0	0.46	26.3	32.6	0.46

Non-obese: BMI < 30.0 kg/m^2^; Obese: BMI ≥ 30.0 kg/m^2^; BMI: body mass index; OPLL: ossification of the posterior longitudinal ligament; COR: canal occupying ratio; ROM: range of motion; T2 high: intramedullary high intensity area on T2-weighted images. Thickness of ossification, occupying ratio, C2-7 angle, and C2-7 ROM were represented by mean ± standard deviation.

**Table 6:** Perioperative complications by each approach.

	Anterior (n = 97)			Posterior (n = 369)		
	Non-obese (n = 82)	Obese (n = 15)	*p*-value	Non-obese (n = 323)	Obese (n = 46)	*p*-value
Complications (%)	39.0	26.7	0.56	21.7	39.1	0.015
**Neurological**						
Quadriparesis (%)	1.2	0.0	1.00	1.9	2.2	1.00
Hemiparesis (%)	1.2	0.0	1.00	0.6	0.0	1.00
Paraparesis (%)	0.0	0.0	–	0.3	0.0	1.00
Upper ext. bil. (%)	1.2	0.0	1.00	0.0	2.2	0.13
Upper ext. uni. (%)	9.8	0.0	0.35	6.2	8.7	0.52
C5 palsy (%)	11.0	0.0	0.35	6.2	8.7	0.52
**Local**						
Dural tear (%)	7.3	20	0.28	3.4	4.3	0.67
CSF leakage (%)	4.9	0.0	1.00	1.2	2.2	0.49
Epidural hematoma (%)	1.2	0.0	1.00	0.3	0.0	1.00
Wound dehiscence (%)	0.0	0.0	–	0.9	2.2	0.41
SSI (%)	1.2	0.0	1.00	1.2	8.7	0.010
Graft complications (%)	9.8	0.0	0.35	0.0	0.0	-
**General**						
Dysphagia (%)	8.5	0.0	0.59	0.6	0.0	1.00
Upper airway obstruction (%)	0.0	0.0	–	0.3	0.0	1.00
PE/DVT (%)	0.0	0.0	–	0.6	2.2	0.33
Delirium (%)	2.4	0.0	1.00	1.5	0.0	1.00
UTI (%)	1.2	0.0	1.00	1.9	4.3	0.26

Non-obese: BMI < 30.0 kg/m^2^; Obese: BMI ≥ 30.0 kg/m^2^; BMI: body mass index; ex: extremity; bil: bilateral; uni: unilateral; CSF: cerebrospinal fluid; SSI: surgical site infection; PE: pulmonary embolism; DVT: deep venous thrombosis; UTI: urinary tract infection.

Neurological improvement was assessed with outcomes 2 years postoperatively. At the time of 2 years after the surgery, 402 patients were available for the analyses, and the follow-up rate was 84.1%. The preoperative total Japanese Orthopedic Association (JOA) scores of the non-obese and obese groups were 10.6 ± 2.9 and 10.4 ± 3.1, respectively, with no significant difference between the two groups (p = 0.63) (Table 7). The total JOA scores of the non-obese and obese groups at 2 years after the surgery were 13.6 ± 2.6 and 13.4 ± 2.5, respectively, showing no significant difference (p = 0.64) (Table 7). The JOA recovery rate of the non-obese and obese groups at 2 years after surgery were 42.2 ± 36.5 and 46.9 ± 32.4, respectively, showing no significant difference (p = 0.32).

**Table 7:** Clinical condition at before and 2 years after surgery in obese and non-obese groups.

	Preoperative JOA score			2-year postoperative JOA score		
	Non-obese	Obese	*p*-value	Non-obese	Obese	*p*-value
**Total**	10.6 ± 2.85	10.4 ± 3.11	0.32	13.6 ± 2.64	13.4 ± 2.51	0.64
Finger motion	2.39 ± 0.91	2.51 ± 1.04	0.35	3.30 ± 0.83	3.28 ± 0.75	0.90
Shoulder-elbow motion	− 0.17 ± 0.43	− 0.20 ± 0.45	0.62	− 0.084 ± 0.32	− 0.061 ± 0.29	0.62
Lower limbs motion	2.18 ± 1.07	2.04 ± 1.14	0.37	2.82 ± 1.08	2.67 ± 1.00	0.32
Upper limbs sensory	0.95 ± 0.47	0.95 ± 0.45	0.98	1.34 ± 0.47	1.37 ± 0.47	0.71
Trunk sensory	1.62 ± 0.59	1.54 ± 0.63	0.40	1.85 ± 0.39	1.83 ± 0.46	0.72
Lower limbs sensory	1.33 ± 0.62	1.31 ± 0.60	0.76	1.64 ± 0.49	1.64 ± 0.48	0.97
BBD	2.35 ± 0.81	2.30 ± 0.80	0.65	2.76 ± 0.53	2.72 ± 0.53	0.63
JOA RR 2Y				46.9 ± 32.4	42.2 ± 36.5	0.32

JOA scores were represented by mean ± standard deviation.

Non-obese: body mass index (BMI) < 30.0 kg/m^2^; Obese: BMI ≥ 30.0 kg/m^2^; BBD: bowel and bladder dysfunction; JOA RR 2Y: Japanese Orthopedic Association recovery rate after 2 years surgery; MCID: minimal clinically important difference.

Overall, 184 out of 402 patients (45.8%) achieved an minimum clinically important difference (MCID) success at 2 years after the surgery. Then, the factors affecting the MCID success were evaluated by logistic regression analysis (Table 8). This analysis revealed that age (OR: 0.946, 95% CI 0.926–0.967, p < 0.001), BMI (OR: 0.906, 95% CI 0.858–0.957, p < 0.001), and duration of symptoms (OR: 0.993, 95% CI 0.989–0.998 p = 0.002) were significant factors affecting the postoperative MCID success. A post hoc power analysis showed that the actual sample size of this study had 99.3% power.

**Table 8:** Factors affecting MCID success.

	OR	95% CI	*p*-value
Age	0.946	0.926–0.967	< 0.001
Gender	0.683	0.420–1.109	0.123
BMI	0.906	0.858–0.957	< 0.001
Duration of symptoms	0.993	0.989–0.998	0.002
Pre JOA score	0.961	0.890–1.037	0.301
COR	1.008	0.994–1.022	0.261

MCID: minimal clinically important difference; BMI: body mass index; JOA: Japanese Orthopaedic Association; COR: canal occupying ratio; OR: odds ratio; CI: confidence interval.

## Discussion

Now, obesity has become a public health concern around the world. In addition, previous studies reported that obese patients are at increased risk of complications such as SSI, deep vein thrombosis, pulmonary embolism, and dural lacerations when undergoing spinal surgery^13–17^. It is therefore no wonder surgeons are worried about the effects of obesity on surgical treatment^17^.

The present study revealed that the frequency of SSI in the obese group (6.15%) was significantly higher than that in the non-obese group (1.21%). This postoperative incidence of SSI in the obese group (6.15%) is around two times higher than the pooled incidence of SSI after spine surgery (3.1%) revealed by a recent systematic review^21^. In the present study, since there was no significant difference between the non-obese and obese groups in comorbidities that were thought to affect perioperative complications such as diabetes mellitus, we considered that the obesity had a significant impact on high SSI incidence. However, we do not immediately deny the possible involvement of factors that did not differ statistically. We believe that the real impact of statistically non-significant factors on SSI will need to be investigated in larger samples in the future.

Approach-specific complication in cervical spine surgery has been suggested^4,7,22^. Anterior surgery tends to have an increased risk for dysphasia, upper airway obstruction, recurrent laryngeal nerve injury, and dural tear. On the other hand, posterior surgery tends to have an increased risk for SSI, postoperative neck pain (axial pain), and postoperative kyphosis. Therefore, this study investigated the effects of obesity on perioperative complications for each surgical approach. Approach-specific analyses disclosed that perioperative complications in the anterior surgery were not significantly different between non-obese and obese groups (Table 6). However, in posterior surgery, the frequency of SSI in the obese group (8.7%) was about seven times higher than that in the non-obese group (1.2%), which was significantly higher (Table 6). Perioperative complications other than SSI in posterior surgery were not significantly different between the two groups. These findings suggested that the significantly higher frequency of SSI in the obese group compared to that in the non-obese group reflected the results of posterior surgery. The anterior approach has the advantages of minimal soft tissue dissection compared to the posterior approach, resulting in a lower incidence of SSI^23^. The surgical procedure for cervical OPLL should be comprehensively determined in careful consideration of cervical spine alignment, COR, K-line, etc., but anterior surgery may be beneficial for patients who are prone to SSI. Alternatively, we should carefully explain that obese patients with cervical OPLL are at high risk of SSI, especially who choose posterior surgery. If neurological compromises allow, it is advisable to consider a preoperative weight loss program aimed at reducing BMI.

In an assessment on postoperative neurological improvement, the JOA recovery rate at 2 years after surgery in the obese group were not significantly different from those in the non-obese group. On the other hand, logistic regression analysis revealed that BMI was one of the significant factors affecting the postoperative MCID success. One of the likely explanations for this discrepancy is the cutoff point that separated non-obese and obese groups. While there are several reports that obesity has negative effects on surgery^13–16^, recent review analyzing the role of obesity on outcomes after spine surgery reported that it's still controversial, but cervical fusion surgery seems to have as valuable results in obese patients as in non-obese patients^17^. However, we must keep in our mind that the results of this review by Cofano et al. have several limitations, that is, fewer citations for papers dealing with cervical spine surgery compared to thoracolumbar spine surgery, the inclusion of cases other than cervical OPLL, and the absence of laminoplasty, which is the most selected treatment for cervical OPLL. Therefore, we believe that no conclusions have been established regarding the actual effect of obesity on postoperative neurological improvement of cervical OPLL, and further studies are needed to clarify it.

We also confirmed a correlation between duration of symptoms and likelihood of MCID success in the present study. The longer the preoperative exacerbation of neurological symptoms, the more irreversible changes can occur in the spinal cord, which can exacerbate postoperative neurological improvement. We believe that this is one of the main causes of the correlation between preoperative duration of symptoms and likelihood of MCID success. However, even if the clinical symptoms are mild, long-term spinal cord compression can have a negative impact on postoperative improvement of the symptom. Thus, when planning a weight loss program for BMI reduction before surgery, we believe that consideration should be given to making it as short as possible.

There were no significant differences between the non-obese and obese groups in the type of cervical OPLL, C2-7 angle, C2-7 ROM, and the presence of an intramedullary intensity changes on T2-weighted MR imaging. However, we found that the COR in the obese group was almost significantly higher than that in the non-obese group (p = 0.050). A previous study revealed that the number of ossified lesions in the whole spine was significantly correlated with obesity^20^. In addition, 3D-imaging analysis using CT reported that OPLL increased volume over the natural course and high BMI was one of the predictors of OPLL progression^24^. Furthermore, previous cross-sectional study revealed that one of the characteristics of young patients with cervical OPLL was high BMI^19^. Consistent with this report, the average age of the obese group in the present study was significantly younger than that of non-obese group. Therefore, obesity may be involved not only in the early onset of cervical OPLL, but also in its progression.

A few systematic reviews or meta-analyses of cervical OPLL reported that the incidence of postoperative complications of cervical OPLL^7,18,25^. It was consistently high compared to other common cervical degenerative diseases (cervical spondylotic myelopathy and cervical disc herniation). The optimal surgical treatment for OPLL has not been established and remain challenging^7,8,18,25^. One of the reasons for this may be the lack of evidence. This study could be an evidence, however further studies need to clarify the real effects of obesity on the cervical OPLL including its treatment and development.

Otherwise, the present study imposes several limitations. This study is a prospective study of surgically treated patients with cervical OPLL, but the surgical procedure has not been randomized. The surgical procedure was determined according to the algorithm shown in the “Patients and methods”. However, in the end, at the discretion of each institution, the surgical procedure for each case was decided. In addition, BMIs of the patients in this study were much lower than expected in other populations such as in North America and Europe. Furthermore, we were able to investigate the effects of obesity on cervical OPLL treatment in short-term outcomes, but not in long-term outcomes. Longer follow-up involving much higher BMI ranges is needed to clarify these issues. However, the favorable aspect of the present study is that this is the first Japanese nationwide, multicenter, prospective study comprehensively disclosing the effects of the obesity on the treatment for patients with cervical OPLL. Furthermore, we investigated the effects of obesity by surgical approaches and clarified for the first time that special attention should be paid to SSI especially in posterior surgery.

In conclusion, this is the first well-powered Japanese nationwide, multicenter prospective study that identified the effect of obesity on the treatment for the patients with cervical OPLL. Special attention should be paid to SSI when planning posterior surgery for the treatment of obese patients with cervical OPLL.

## Patients and methods

### Participants

This Japanese nationwide, multicenter, prospective study (https://center6.umin.ac.jp/cgi-open-bin/ctr/ctr_view.cgi?recptno=R000039771, UMIN000035194) involved 28 academic institutions of the Japanese Multicenter Research Organization for Ossification of the Spinal Ligament (JOSL) formed by the Japanese Ministry of Health, Labour and Welfare and the Japanese Agency for Medical Research and Development (AMED). The protocol for this study has been approved by institutional review board (IRB) of Tokyo Medical and Dental University as a Central IRB and IRB of Shiga University of Medical Science and the other all participating institutions as a local participating institution. This research was conducted in accordance with the "Declaration of Helsinki" and the Ministry of Health, Labor and Welfare/Ministry of Education, Culture, Sports, Science and Technology "Ethical Guidelines for Medical Research for Humans".

In total, 478 patients (354 males, 124 females, mean age 64.1 ± 11.6 years) with cervical myelopathy due to OPLL were prospectively enrolled from April 2015 to July 2017. The definition of cervical myelopathy due to OPLL is that (1) clear spinal cord compression by OPLL can be confirmed, and (2) spinal cord compression by OPLL can be diagnosed as the cause of the present symptoms by neurological examination. Data were collected from each patient, including demographic information, medical history, and clinical and radiographical findings. Patients with (i) less than 20 years old; (ii) a history of cervical spine surgery; (iii) neurological impairments due to cervical disc herniation, infection, or trauma were excluded from this study. All patients provided written informed consent on entry into the registry.

The surgical procedure was determined according to the following algorithm. In general, cervical laminoplasty was selected for K-line (+) cases with the lordotic alignment. Contrary, in patients with K-line (−) cases and/or high spinal canal occupying ratio of OPLL and patients with kyphosis, anterior decompression and fusion and/or posterior decompression and fusion were employed. However, anterior surgery is a technical demand surgery with higher incidence of complications and was adopted after comprehensively evaluating the experience of the surgeon and the wishes of the patient.

To clarify the effects of obesity on the surgical treatment for cervical OPLL, patients were stratified into two groups, non-obese (< BMI 30.0 kg/m^2^) and obese (≥ BMI 30.0 kg/m^2^) groups according to the WHO classification^9^. It has been reported that different complications occur with different surgical approaches in the treatment of cervical spine disorders^4,7,22^. Thus, to clarify the effects of obesity on this issue, we divided the surgery into anterior (anterior decompression and fusion) and posterior (laminoplasty and posterior decompression and fusion) surgeries. Patients who underwent anterior–posterior surgery were excluded in this approach-specific analyses.

### Radiographical assessments

The types of OPLL were categorized as segmental-, continuous-, mixed-, and localized-type^26^. Sagittal alignment of the cervical spine defined by the Cobb angle between C2 and C7 (C2-7 angle) and K-line (+/−)^27^ were determined on a lateral standard radiograph in the neutral position. The K-line is a straight line connecting the midpoints of the spinal canals of C2 and C7 in the lateral radiograph. Patients were considered K-line (−) if OPLL crossed the K-line, and K-line (+) otherwise. C2-7 range of motion (ROM) was measured on flexion–extension lateral standard radiographs. The most compressed level and the presence of a signal intensity change in the spinal cord were also investigated on T2-weighted magnetic resonance (MR) imaging. Spinal canal occupying ratio (COR) of OPLL on axial computed tomography (CT) at the maximum cord compression level was also investigated.

### Clinical assessments

The pre- and postoperative clinical condition of the participants was determined using the Japanese Orthopedic Association (JOA) score (maximum 17 points) for cervical myelopathy^28^. The JOA recovery rate was calculated based on the Hirabayashi’s method as follows: (postoperative JOA − preoperative JOA)/(17 − preoperative JOA) × 100^29^. The minimum clinically important difference (MCID) of the JOA recovery rate was defined as 52.8% according to the previous report^30^.

Perioperative complications were defined as any adverse event arising intraoperatively or within 30 days of surgery and were prospectively recorded. Postoperative motor disturbance was defined as at least one grade lower of preoperative strength in key muscle groups via manual muscle testing. Transient deterioration of muscle strength was also included in this definition. We defined SSI, according to the criteria of the Centers for Disease Control and Prevention^31^, as a condition resulting in an abscess or other evidence of infection in the skin, subcutaneous tissue, or deep soft tissue. In addition, the presence of SSI was confirmed by reoperation or by histopathological or radiographical investigation.

### Statistical analyses

To measure the differences between the two groups, we used the Chi-square test and Fisher’s exact test for categorical variables, and the t-test and Welch’s test for continuous variables. Logistic regression analysis was performed to evaluate the factors that affected the success in achieving the MCID of JOA recovery rate. *P* < 0.05 was considered as statistically significant. A post hoc power analysis was performed using an α of 0.05, which was set according to previous studies^32,33^. The software application used for the analysis was SPSS version 26.0 (SPSS Inc., Chicago, IL).
